# Association between Ability Emotional Intelligence and Left Insula during Social Judgment of Facial Emotions

**DOI:** 10.1371/journal.pone.0148621

**Published:** 2016-02-09

**Authors:** Tiziana Quarto, Giuseppe Blasi, Chiara Maddalena, Giovanna Viscanti, Tiziana Lanciano, Emanuela Soleti, Ivan Mangiulli, Paolo Taurisano, Leonardo Fazio, Alessandro Bertolino, Antonietta Curci

**Affiliations:** 1 Psychiatric Neuroscience Group, Department of Basic Medical Sciences, Neuroscience and Sense Organs, University of Bari “Aldo Moro”, Bari, Italy; 2 Cognitive Brain Research Unit, Institute of Behavioral Science, University of Helsinki, Helsinki, Finland; 3 Department of Education Science, Psychology and Communication Science, University of Bari "Aldo Moro", Bari, Italy; 4 pRED, NORD DTA, Hoffman-La Roche Ltd, Basel, Switzerland; University of Bologna, ITALY

## Abstract

The human ability of identifying, processing and regulating emotions from social stimuli is generally referred as Emotional Intelligence (EI). Within EI, Ability EI identifies a performance measure assessing individual skills at perceiving, using, understanding and managing emotions. Previous models suggest that a brain “somatic marker circuitry” (SMC) sustains emotional sub-processes included in EI. Three primary brain regions are included: the amygdala, the insula and the ventromedial prefrontal cortex (vmPFC). Here, our aim was to investigate the relationship between Ability EI scores and SMC activity during social judgment of emotional faces. Sixty-three healthy subjects completed a test measuring Ability EI and underwent fMRI during a social decision task (i.e. approach or avoid) about emotional faces with different facial expressions. Imaging data revealed that EI scores are associated with left insula activity during social judgment of emotional faces as a function of facial expression. Specifically, higher EI scores are associated with greater left insula activity during social judgment of fearful faces but also with lower activity of this region during social judgment of angry faces. These findings indicate that the association between Ability EI and the SMC activity during social behavior is region- and emotion-specific.

## Introduction

Social behavior crucially characterizes humans, involves several aspects of human functioning [1, 2] and is linked with emotional attributes of intelligence [3]. These attributes have been conceptualized in the framework of Emotional Intelligence (EI) [4, 5], which integrates aspects of emotional information processing, emotion regulation and behavioral responses to emotional stimuli [6]. In particular, the ability to process one’s own and others’ emotions is an essential feature of EI.

Two broad models, described as “mixed/trait” models or “ability” models, have been developed in order to conceptualize EI. The first defines EI as an eclectic mix of emotion-related qualities together with personality, motivation and affective dispositions [7], and it has been assessed by self-report measures [8]. The second conceptualizes EI as a form of intelligence dealing with emotions and the processing of emotional information [4], and it has typically been assessed by maximal-performance measures, similar to assessment of the intelligence quotient. This latter model (Ability EI) is particularly intriguing because it is based on the crucial role of the social context in substantiating EI abilities. Furthermore, the conceptualization of EI as a form of intelligence/ability allowed the development of training programs focusing on emotional correlates of mental health and aiming at improving individuals’ well-being [9–11]. Consistently, previous studies have suggested that Ability EI predicts job performance and academic achievements [12–14], as well as positive social interaction [15], mental health [16] and well-being [17], while impaired or deficient Ability EI has been linked to substance abuse [18], anxiety and depression [19].

Less is known about brain correlates of EI. Previous models [20] suggest that a brain “somatic marker circuitry” (SMC) sustains emotional sub-processes included in EI [20–22]. This neuronal circuit consists of three primary brain structures:—the amygdala, which triggers initial signals of emotional salience in response to a relevant stimulus;—the insula, which contributes to the “feeling” of emotion by neurally mapping these somatosensory and visceral sensations, which can be later simulated within the brain when a comparable stimulus is encountered in the future;—the ventromedial prefrontal cortex (vmPFC), which integrates emotional signals with cognitive representations.

Consistent with a putative relationship between EI and SMC, lesion studies have indicated that patients with damaged vmPFC, amygdala or insula present severe impairments in social decision making [21, 23–27]. Furthermore, Bar-On and colleagues [20] have also reported that patients with lesions of the vmPFC, amygdala or insular cortex present significantly lower levels of EI compared with patients presenting lesions in other brain regions. Neuroimaging results on healthy individuals also support the association between SMC and EI [28–34], although only a small number of these studies investigates EI as Ability. For example, one study has found that measures of Ability EI are positively related to gray matter volume of the vmPFC and insula [32]. Moreover, other studies have investigated functional brain activity during tasks measuring aspects of social processing, including processing of facial expressions, which is key for each component of Ability EI [35, 36]. In this context, previous findings indicated that vmPFC activation during a social reasoning task was negatively correlated with measures of Ability EI [28]. Furthermore, another study reported a positive correlation between Ability EI and activity of SMC regions (particularly vmPFC) in response to faces with decreasing level of trustworthiness [33]. The task used in this latter study required the passive viewing of faces, without any social judgment or decision making. However, the EI construct indicates social decision making and evaluation of facial expressions as two crucial components of emotional processing and social interactions [21, 37]. To our knowledge, no study has so far investigated the brain correlates of Ability EI during a social task eliciting both these components.

The aim of this study is to investigate with functional magnetic resonance imaging (fMRI) the relationship between Ability EI scores and brain activity during social behavior involving emotional facial evaluation and decision making. Specifically, we hypothesized that Ability EI scores would predict activity in SMC regions during social judgment of facial expressions.

## Materials and Methods

### Ethics Statement

The present study was approved by the Comitato Etico Indipendente Locale of the Azienda Ospedaliera ‘‘Ospedale Policlinico Consorziale” of Bari. Informed written consent was obtained from all participants before participation to the study.

### Subjects

Sixty-three subjects (34 females; mean ± standard deviation (SD), 29.4±6.3 years), recruited by word of mouth, participated to the study. Inclusion criteria were absence of any psychiatric disorder, as evaluated with the Structured Clinical Interview for Diagnostic and Statistical Manual of Mental Disorders IV, of any significant neurological or medical condition revealed by clinical and magnetic resonance imaging evaluation, of history of head trauma with loss of consciousness, and of pharmacological treatment or drug abuse in the past year. The Wechsler Adult Intelligence Scale–Revised was used to evaluate the Intelligence Quotient (IQ) (mean ± SD, 112 ± 12.3), the Edinburgh Inventory [38] to measure handedness (mean±SD, 0.8±0.4) and the Hollingshead Four Factor Index [39] to measure socio-economic status (mean±SD, 41.8±16.7) (Table 1). All subjects completed the EI test and underwent fMRI during a social judgment task with different facial expressions. The present experimental protocol was approved by the local institutional review board. After a complete description of the study was given to the subjects, written informed consent was obtained.

**Table 1:** Demographics data.

	Age (years)	Females	Hollingshead	Handedness	IQ
Subjects (*n* = 63)	29.4 (6.3)	34	41.8 (16.7)	0.8 (0.4)	112 (12.3)

Demographics of the subjects included in the study. Data are presented as mean and standard deviation.

### Emotional Intelligence Test

To measure Ability EI, each participant completed the validated Italian version [40] of the Mayer–Salovey–Caruso Emotional Intelligence Test (MSCEIT) [37], which includes 141 self-administered items to assess individual skills at perceiving, using, understanding and managing emotions. The MSCEIT yields an *EI Total* score and two sub-scores, *Experiential EI* and *Strategic EI*. High scores on Experiential EI indicate proneness to perceive emotions and effectiveness in using emotional information to facilitate thought and performance. This area includes two subscales measuring abilities described as *Perceiving* and *Using* emotions. The second area is Strategic EI. High scores on this area implicate excellent capacity in understanding emotional information and in managing emotions of themselves and of others. Strategic EI comprises two subscales measuring abilities described as *Understanding* and *Managing* of emotions. MSCEIT scoring was based on the consensus scoring methods outlined in the manual [37].

### fMRI Experimental Paradigm

The event-related fMRI paradigm used in this study has been described in previous neuroimaging studies [41–44]. Briefly, we presented angry, fearful, happy, and neutral facial expressions from a validated set of facial pictures (NimStim,http://www.macbrain.org/resources.htm) [45]. The order of stimuli was randomly distributed. However, every subject saw the same stimuli with the same exact order. During the task, subjects had to decide whether they would like to “approach” or “avoid” the face. From stimulus appearance, 2 s were allowed for behavioral responses. Each stimulus was presented for 500 ms, with the interstimulus interval randomly jittered between 2 and 7 s (ISI average after jittering: 2.7 s). The total number of stimuli was 144: 30 angry, 39 fearful, 37 happy, and 38 neutral faces. The duration of the task was 6 min 8 s. A fixation crosshair was presented during the interstimulus interval.

### Behavioral Data Analysis

Factorial regression analyses were conducted using behavioral data (approach/avoidance evaluation and reaction time at the social judgment task) as dependent variables, Facial Expression as within-subjects categorical predictor and EI total score as between-subjects continuous predictor. In order to avoid the confounding effects of demographic variables, Pearson’s correlation analyses were used to investigate the relationship between EI scores and continuous demographic variables (Age, IQ, Hollingshead, Handedness), while ANOVA was used to investigate the effect of gender on EI scores. Using these analyses, gender resulted to be significantly associated with EI total score. Thus, this variable was used as a covariate of no interest in all the following analyses of this study.

### fMRI Data Acquisition

FMRI was performed on a GE Signa 3T scanner with a gradient echo-planar imaging sequence (repetition time, 2000 ms; echo time, 28 ms; 26 interleaved slices, thickness of 4 mm, gap of 1 mm; voxel size, 3.75 X 3.75 X 5; scan repetitions, 180; flip angle, 90°; field of view, 24 cm; matrix, 64x64). The first four scans were discarded to allow for signal saturation. The visual stimuli were presented via a back-projection system using the stimulation software Presentation (Version 9.00, Neurobehavioral Systems, Albany, CA, USA). A fiber optic response box was used to measure subject preference (and reaction time) for each stimulus: left button for the “approach” response and right button for the “avoid” response.

### fMRI Data Analysis

Analysis of the fMRI data was completed using Statistical Parametric Mapping (SPM8; http://www.fil.ion.ucl.ac.uk/spm). For each subject, images were realigned to the first volume in the time series and movement parameters were extracted to check for excessive head motion (> 2 mm of translation, > 1.5° rotation). Images were then re-sampled to a 3.75 mm isotropic voxel size, spatially normalized into a standard stereotactic space (Montreal Institute on Neurology, MNI, template) and smoothed using a 8 mm full-width half-maximum isotropic Gaussian kernel to minimize noise and to account for residual inter-subject differences. FMRI responses were modeled using a canonical hemodynamic response function (HRF) at each voxel. Vectors were created for happy, angry, fearful and neutral faces. Six subject-specific movement parameters obtained after the realignment procedure were included in the model as covariates of no interest in order to control for potential effects of motion. Predetermined condition effects at each voxel were created using a t statistic, producing a statistical image for BOLD responses to brain processing of stimuli representative of each condition, i.e., happy, angry, fearful and neutral faces versus fixation crosshairs. Therefore, individual contrast estimates were entered in a second-level factorial regression analysis using Facial Expression as the within-subjects predictor and EI total score as the continuous factor. Furthermore, in order to investigate the effect of each subscale of EI, four separate regression models were created with Perceiving, Facilitating, Understanding and Managing as the continuous predictors. All the analyses were constrained by a mask obtained by combining group activation maps associated with processing of each facial expression, in order to focus our analyses on brain regions activated during the task compared to the baseline. We used a statistical threshold of a Family Wise Error (FWE) voxel-level corrected p < 0.05 (minimum cluster size [k] = 10), using as volume of interest three separate masks including the Wake Forest University Pickatlas (http://fmri.wfubmc.edu/software/PickAtlas) bilateral ventromedial prefrontal cortex (vmPFC), bilateral amygdala, and bilateral insula. These regions were chosen a priori based on earlier lesion and neuroimaging studies investigating the neurological substrates of EI [21, 24, 33]. A Bonferroni correction for the number of investigated regions (N = 3) was also applied. In order to control for type II errors, we also investigated not hypothesized potential effects outside our ROI. For such investigation, we used a statistical threshold of a FWE whole brain corrected p < 0.05 (minimum cluster size [k] = 10), since we did not have a priori hypothesis on areas outside the SMC. T-contrasts were used as post-hoc analyses to determine statistical differences between regression slopes and statistical significance of each regression slope. BOLD responses were extracted from significant clusters using MarsBar (http://marsbar.sourceforge.net/) for illustrative purposes.

## Results

### Behavioral Data

Pearson’s correlation did not reveal significant correlations between EI total score and continuous demographic variables (Age, IQ, Hollingshead, Handedness) (all r<0.1; p>0.2). Furthermore, ANOVA indicated that EI total score was significantly associated with gender, with females having greater EI total score than males (F_(1, 61)_ = 4.46; p = 0.038). Thus, gender was used as a nuisance variable in all the following analyses. Factorial regression analyses revealed that RT and number of approach/avoidance judgments were not significantly predicted by EI total score or by the interaction between EI total score and Facial Expression (all p>0.2) (detailed results of these analyses are reported in the Supporting Information (S1 File).

### fMRI Data

**Fig 1:**
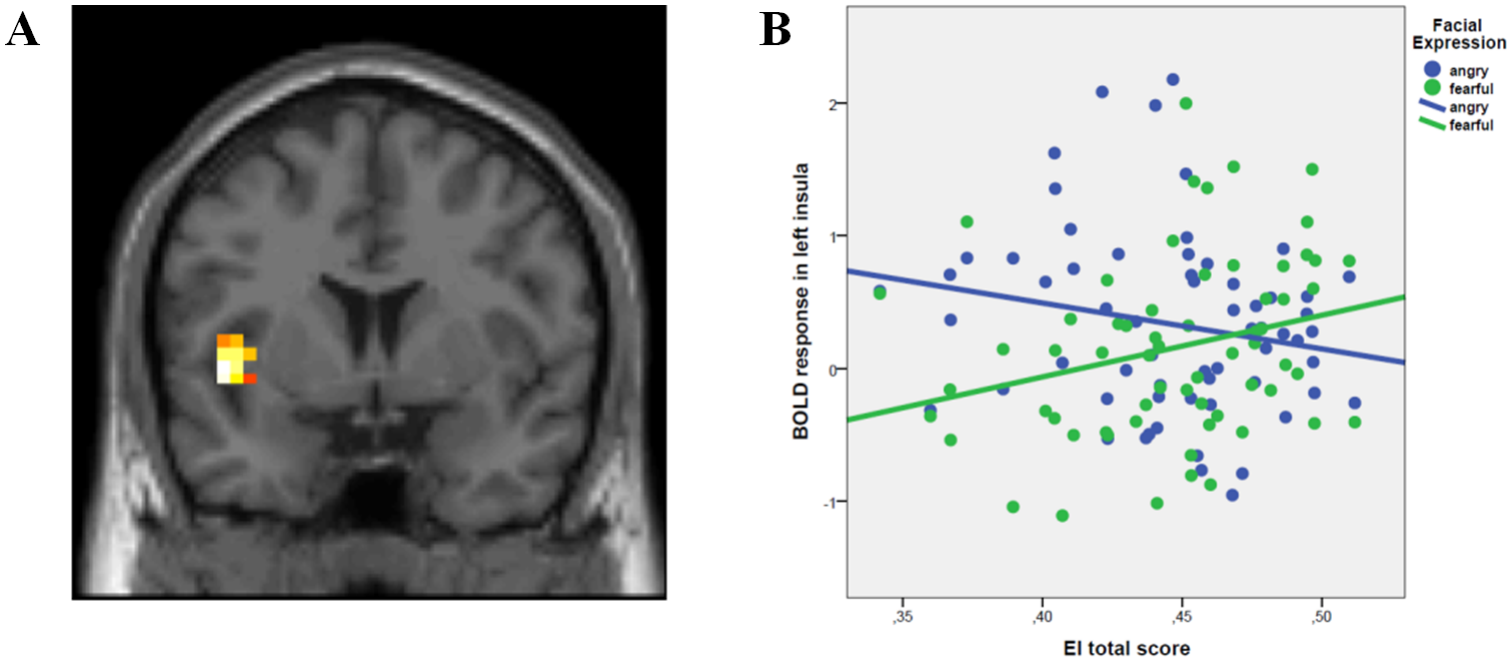
**A.** Coronal section showing the interaction between EI total score and Facial Expression in left insula. **B.** Scatterplot depicting the relationship between EI and activity in left insula during social judgment of fearful faces and angry faces. See text for statistics.

Multi-factorial regression analyses demonstrated no significant effect of EI total score, while there was an effect of Facial Expression in right vmPFC (x = 34 y = 46 z = 29; K = 19; Z = 4.04; FWE-p = 0.009), left insula (x = -26 y = 8 z = -16; K = 101; Z = 4.23; FWE-p = 0.006) and bilateral amygdala (x = 27 y = 0 z = -20; K = 21; Z = 5.54; FWE-p<0.001; x = -26 y = 0 z = -20; K = 20; Z = 4.49; FWE-p<0.001). In particular, post-hoc T-contrasts in these brain regions revealed that processing of happy, angry and fearful faces was associated with greater activity than that of neutral faces. Furthermore, processing of happy and angry faces had greater activity than that of fearful faces (all p<0.05). Moreover, we found an interaction between EI total score and Facial Expression in left insula (x = -46 y = 4 z = -1; K = 32; Z = 3.4; FWE-p = 0.03) (Fig 1A). Post-hoc *t*-tests revealed that this interaction was explained by a statistically different relationship between EI total score and insula activity during fearful compared with angry faces (p<0.001). Specifically, higher scores of EI predicted greater insula activity during social judgment of fearful faces (p = 0.006) but lower insula response during social judgment of angry faces (p = 0.04) (Fig 1B). No other significant effects were found within and outside the hypothesized ROI.

Finally, separate multi-factorial regression analyses demonstrated lack of effect of EI subscales or interaction between EI subscales and Facial Expression on brain activity.

## Discussion

The present study examined the relationship between EI, as measured by an ability-based test (MSCEIT) [37, 40] and activity of brain regions within the SMC during social judgment of facial expressions of emotions. Consistent with previous findings [20, 46–49], our ROI-based investigation indicated that vmPFC, insula and amygdala were significantly involved in the social judgment of emotional faces. Moreover, our results indicate that EI total score is associated with left insula activity during social judgment of emotional faces as a function of facial expression. Specifically, greater EI total score predicted greater left insula activity during social judgment of fearful faces but lower activity of this region during social judgment of angry faces. These results are consistent with previous findings indicating a double dissociation between individual personality features and neural response to angry and fearful faces [50]. In particular, Ewbank et al. [51] found that anxiety predicted a greater amygdala response to angry compared to fearful faces for attended facial expressions. Differently, anxiety predicted a greater amygdala response to fearful than angry faces for unattended facial expressions. According to previous models [52], the authors of this study suggest that an interpretation of these findings relies on the evidence that angry and fearful faces represent qualitatively different forms of threat. Fearful faces are thought to signal the presence of a significant, yet undetermined source of danger within the environment, referred to as ‘ambiguous threat’. On the other hand, angry faces represent a more direct form of threat, often used in face-to-face encounters to exert dominance. Consistently, previous reports have also highlighted that anger and fear produce qualitatively different bodily reactions [53]. In this line of reasoning, our findings suggest that EI may modulate the relationship between left insula activity and processing of different emotional information and bodily reactions conveyed by angry vs. fearful faces.

Insular cortex is one of the key regions within the SMC [21]. Since the original formulation of the Somatic Marker Hypothesis, the insula has been considered a crucial brain region for the bodily feeling of emotion [24]. Accordingly, this area has been defined a key area for ‘‘interoceptive awareness”, i.e. the ability to correctly perceive all bodily signals and efficiently integrate them in a single emotional framework, through connections with other neural sources [54]. Consistent with this contention, a growing body of literature has also suggested the role of the insula in meditation practice [55–57], for which interoceptive and emotional awareness appear to be crucial aspects [55, 56]. Based on this literature, our results may be interpreted in a conceptual framework in which the emotional intelligence may be linked with the ability to integrate different emotional signals mediated by the insular cortex.

In the present study, we found a left lateralized interaction between Ability EI and Facial Expression in the insula. This finding is consistent with a recent meta-analysis reporting a hemispheric asymmetry of emotional processing in the insula based on the quality of the emotional stimulus as well as on the emotional processing required [58]. In particular, this study reports left-lateralized insula activity during emotional perception and experience. Thus, it is possible that the task used in our study requires emotional processes more linked with a left lateralized activity of the insula.

Of note, in the present study we did not find any significant effect of the specific subscales of EI on the activity of SMC, suggesting that the subcomponents of EI are together but not individually predictive of the differential insular activity during social judgment of fearful and angry faces. Whether this is in line with a previous fMRI study that used the same measure of Ability EI [28], we cannot exclude that our study was not powered to detect association of neurobiological correlates with more subtle constructs of EI. Indeed, lack of adequate statistical power also prevented us to investigate the interaction of EI subscales on processing of facial expressions.

To date, only one fMRI study explored the relationship between Ability EI and activity of brain regions within the SMC during emotional faces processing. The authors of this study failed to find correlations between EI and insula activity, whether they found a correlation between EI and vmPFC activity [33]. However, emotional stimulation in this study was obtained by an implicit emotional task consisting in passive vision of faces with different level of trustworthiness, while our paradigm implicates explicit evaluation of emotional faces, which may explain lack of consistency of results. Importantly, facial evaluation and social decision making are two crucial aspects of EI [21, 37].

Importantly, in our study we did not find any association between EI and behavioral response to the neuropsychological task. This finding suggests that fMRI brain activity during our task is more linked to EI compared to the behavioral correlate that we measure here. In general, it also suggests that the power of detection associated with the investigation of imaging phenotypes is much greater compared to those implicated by the study of behavioral correlates. Indeed, this contention is consistent with several previous studies indicating association of a given predictor with fMRI activity, but not with behavior [59, 60].

A limitation of this study is that we did not counterbalance response alternatives at the task between subjects. However, behavioral data indicated that the approach/avoidance choice did not significantly affect subjects’ reaction times (F_(1,60)_ = 0.50; p = 0.48). Furthermore, the purpose of our study was not to investigate brain activity associated with processing of avoided vs. approached facial expressions. Thus, we believe that this limitation might not have biased the main findings of the present study.

In conclusion, the present results indicate that EI differentially predicts left insula activity during social judgment of fearful and angry faces. These findings may help uncovering the link between EI and the neural correlates of basic emotions and may represent an important step towards the understanding of neural mechanisms behind psychiatric disorders that implicate alterations in social and emotional functioning.

## Supporting Information

S1 File Behavioral Data. (PDF)
