# Supplementary material for: Association between Ability Emotional Intelligence and Left Insula during Social Judgment of Facial Emotions
Source: PLoS One. 2016 Feb 9;11(2):e0148621. doi: 10.1371/journal.pone.0148621 (PMC4747486; doi:10.1371/journal.pone.0148621)
Supplement: S1 File — (PDF) [file pone.0148621.s001.pdf]

Behavioral Results

Factorial regression analyses were performed using behavioral data (reaction times and approach/avoidance evaluation at the social judgment task) as dependent variables, Facial Expression as within-subjects categorical predictor and EI total score as between-subjects continuous predictor. These analyses revealed an effect of Facial Expression on RT ($F_{(3, 186)}=15.77$; $p<0.001$). Fisher's post-hoc test indicated slower RT during social judgment of neutral faces compared to all the other faces and faster RT during social judgment of angry and fearful faces compared to happy and neutral faces (all $p<0.05$). No effect of EI total score or an interaction between EI total score and Facial Expression were found on this behavioral measure (all $p>0.2$).

Furthermore, there was an effect of Facial Expression on the number of approach/avoidance judgments ($F_{(3, 186)}=176.83$; $p<0.001$). Here, post-hoc analysis revealed greater avoidance for fearful compared to the other facial expressions ($p<0.001$). Furthermore, angry faces were more avoided than happy and neutral faces ($p<0.001$). Moreover, there was greater avoidance for neutral compared to happy faces ($p<0.001$). Finally, number of approach/avoidance judgments were not significantly predicted by EI total score or by the interaction between EI total score and Facial Expression (all $p>0.2$).
